# Distinct Mechanical Properties of the Respiratory System Evaluated by Forced Oscillation Technique in Acute Exacerbation of COPD and Acute Decompensated Heart Failure

**DOI:** 10.3390/diagnostics11030554

**Published:** 2021-03-19

**Authors:** Silvia Terraneo, Rocco Francesco Rinaldo, Giuseppe Francesco Sferrazza Papa, Fulvia Ribolla, Carlo Gulotta, Laura Maugeri, Emiliano Gatti, Stefano Centanni, Fabiano Di Marco

**Affiliations:** 1 Respiratory Unit, ASST Santi Paolo e Carlo, San Paolo Hospital, Department of Health Sciences, Università degli Studi di Milano, 20142 Milan, Italy; silvia.terraneo@asst-santipaolocarlo.it (S.T.); stefano.centanni@unimi.it (S.C.); 2 Department of Health Sciences, Università degli Studi di Milano, 20142 Milan, Italy; francesco.sferrazza@unimi.it; 3 Cardiovascular and Thoracic Department, SC Pneumologia U, Città della Salute e della Scienza (Molinette) University Hospital, 10126 Turin, Italy; 4 Pneumologia AOU San Luigi Gonzaga, 10043 Orbassano, Turin, Italy; fulvia.ribolla@gmail.com; 5 Respiratory Unit, Papa Giovanni XXIII Hospital, Department of Health Sciences, Università degli Studi di Milano, 24127 Bergamo, Italy; c.gulotta@sanluigi.piemonte.it (C.G.); l.maugeri@sanluigi.piemonte.it (L.M.); e.gatti@sanluigi.piemonte.it (E.G.); 6 Casa di Cura del Policlinico, Department of Neurorehabilitation Sciences, 20144 Milan, Italy; fabiano.dimarco@unimi.it

**Keywords:** heart failure, acute exacerbation of COPD, FOT

## Abstract

Discriminating between cardiac and pulmonary dyspnea is essential for patients’ management. We investigated the feasibility and ability of forced oscillation techniques (FOT) in distinguishing between acute exacerbation of COPD (AECOPD), and acute decompensated heart failure (ADHF) in a clinical emergency setting. We enrolled 49 patients admitted to the emergency department (ED) for dyspnea and acute respiratory failure for AECOPD, or ADHF, and 11 healthy subjects. All patients were able to perform bedside FOT measurement. Patients with AECOPD showed a significantly higher inspiratory resistance at 5 Hz, Xrs_5_ (179% of predicted, interquartile range, IQR 94–224 vs. 100 IQR 67–149; *p* = 0.019), and a higher inspiratory reactance at 5 Hz (151%, IQR 74–231 vs. 57 IQR 49–99; *p* = 0.005) than patients with ADHF. Moreover, AECOPD showed higher heterogeneity of ventilation (respiratory system resistance difference at 5 and 19 Hz, Rrs_5-19_: 1.49 cmH_2_O/(L/s), IQR 1.03–2.16 vs. 0.44 IQR 0.22–0.76; *p* = 0.030), and a higher percentage of flow limited breaths compared to ADHF (10%, IQR 0–100 vs. 0 IQR 0–12; *p* = 0.030). FOT, which resulted in a suitable tool to be used in the ED setting, has the ability to identify distinct mechanical properties of the respiratory system in AECOPD and ADHF.

## 1. Introduction

Acute exacerbation of chronic obstructive pulmonary disease (AECOPD) and acute decompensated heart failure (ADHF) are two of the most frequent clinical conditions that occur in patients presenting to the emergency department (ED) with dyspnea and respiratory failure [1,2]. Because of the high prevalence of their underlying chronic disease such as chronic obstructive pulmonary disease (COPD) and chronic heart failure (CHF), the epidemiological burden of such conditions is remarkable. Moreover, due to the concomitant risk factors for both COPD and CHF (e.g., tobacco smoking, age, etc.), the two diseases can coexist in a significant number of patients [3,4,5,6]. To establish the correct diagnosis in ED is essential in order to set up the appropriate clinical management. Different procedures have been proposed so far to aid in differentiating cardiac or pulmonary dyspnea in ED (e.g., point of care thoracic ultrasound, pro-brain-type natriuretic peptide) since the clinical presentation of both AECOPD and ADHF is characterized by such symptom. Nevertheless, differential diagnosis remains a notable clinical challenge in some cases [2], thus, the evaluation of other tools useful in this context is of great medical interest.

The mechanical properties of the lung are expected to be different in the case of CHF and COPD, with a respiratory system characterized by low compliance and normal/slightly high airway resistances in the first case, and high lung compliance (at least in part balanced by the high volume of the respiratory system due to lung hyperinflation) and markedly high airways resistances in the latter [7,8,9]. The forced oscillation technique (FOT) is a tool for the investigation of respiratory mechanics introduced in 1956 and constantly improved since [10]. The major advantage of the FOT is being noninvasive and requiring minimal cooperation from the patients, thus making it an important resource in different clinical setting, including pediatric patients. So far, FOT has been used in several conditions [11,12,13], mainly to describe the severity of airway obstruction [14] or to detect the response of the airways to bronchodilators [15], with both research and clinical purposes. An in-vivo study carried out by Dellacà et al. in 2008 showed that at very early stages of interstitial edema could significantly affect the mechanical features of lung tissue measured with FOT [16].

The aims of the present study were to investigate whether FOT is a feasible tool in an emergency setting, and whether this could be an aid to distinguish the mechanical properties of the respiratory systems in patients presenting with AECOPD or ADHF.

## 2. Materials and Methods

This was an observational multicentre prospective study carried out at the Respiratory Unit and Emergency Department of San Paolo University Hospital (ASST Santi Paolo e Carlo, Milan, Italy) and at the Respiratory Unit of San Luigi Gonzaga Hospital in Orbassano (Turin, Italy). Final approval was granted by the Milan Area A Ethical Committee (principal site) with registration number 80/ST/2014, and written consent was obtained from each participant. No extramural funding was used to support the study.

### 2.1. Population in Analysis

Three groups of subjects were included in the study: (1) AECOPD, (2) ADHF, and (3) healthy volunteers, representing the control group (Table 1). Consecutive patients presented at ED for dyspnoea and acute respiratory failure (PaO_2_ < 60 mmHg breathing room air) and diagnosed with an acute deterioration of their chronic disease that required a stronger pharmacological approach and low flow oxygen therapy. Patients were recruited if known for: (1) a previous diagnosis of chronic heart failure according to ESC guidelines both with reduced and preserved ejection fraction [17], without pulmonary obstructive disease (investigated at least by spirometry), or other chronic lung disease, without any instrumental or clinical sign suggesting concurrent pulmonary involvement; (2) patients with a previous COPD diagnosis according to ATS/ERS guidelines (post-bronchodilator FEV_1_/FVC <0.7 with a smoking or exposure history consistent with the diagnosis) [18], without any previous diagnosis of chronic heart failure (investigated at least with echocardiography), and without the evidence of a cardiac involvement during the evaluation in the emergency room; (3) control group: asymptomatic subjects, never smokers or former smokers with a smoking history <10 pack-years and a normal spirometry (FEV_1_/FVC, FEV_1_, and FVC > LLN), sex and age matched with the study population. Patients were recruited during ED department stay and in the Respiratory and Cardiology Clinic of San Paolo Hospital (Milan, Italy) or Respiratory and Cardiology Unit of San Luigi Gonzaga Hospital in Orbassano (Turin, Italy), whilst healthy subjects were enrolled from the local community. Patients in both group 1 and group 2 were regularly followed-up, respectively in the cardiology and respiratory clinic of the two hospitals. Patients were excluded if unstable, if required ventilatory support such as high flow nasal cannula, CPAP, noninvasive or invasive ventilation, or if requiring critical care. Patients were also excluded if there was evidence of another acute respiratory condition or evidence of acute myocardial infarction or if the patient’s conditions required invasive management. Participation in the study did not influence the treatment of patients.

### 2.2. Study Design

The experimental session was performed at the patient’s bedside during ED stay. After written consent, all patients underwent a blood test, including pro-BNP measurement, and blood gas analysis. Medical history, clinical signs, and physical examination at presentation in the ED were recorded. All patients performed the assessment of respiratory mechanics during spontaneous breathing by FOT (Resmon Pro Restech, Milan, Italy) with a multiple frequency 5–11–19 Hz protocol. The test was performed while the patients were seated and breathing at tidal volume, according to ERS guidelines [19]. At least three technically acceptable measurements (10 breaths each) were obtained, thus requiring about 20–25 s per single measurement. The presence of expiratory flow limitation (EFL) was defined as the percentage of total breath with ΔXrs (respiratory system reactance) >2.8 cmH_2_O/(L/s) [20].

### 2.3. Feasibility and Safety Procedures

All the measures of the study were taken under strict medical monitoring by an investigator of the study (all medical doctors), including continuous SpO2. All the investigators were instructed to stop the procedure immediately in case of any sign of deterioration of patient clinical status or per patients’ request. In addition, an interval of up to 30 min from one measurement to another was taken into account in the protocol. Evaluation of the feasibility of FOT in the clinical scenario was a secondary outcome of the study.

### 2.4. Statistical Analysis

We computed that a sample size of 18 patients per arm was needed to consider an expected difference of one standard deviation between the group of AECOP and ADHF patients, with the latter being less flow limited, with a power of 90% and an α error of 5%, based on the paper from Dellacà et al. [21], who reported a mean value of 3.58 (3.33) for the ΔXrs from a group of COPD patients. With an expected rate of drop out or data missing of 20% we decided to enroll at least 22 patients per arm. The results are shown as median and interquartile range unless otherwise stated. Lilliefors corrected K-S test was performed before the data analysis in order to examine the distribution of the residuals of the parametric tests. Quantitative variables were analyzed using T-test or Mann–Whitney when appropriated and analysis of variance (ANOVA), or Kruskal–Wallis test when appropriate. In the case of *p* < 0.05, follow-up analyses were carried out by pairwise comparisons using Bonferroni adjustment. For qualitative variables, either chi-square or a Fischer exact test was used. We developed a receiver operating characteristic (ROC) curve, to detect the optimal cut off point at which the sensitivity and specificity for AECOPD of every clinically relevant FOT parameter were maximized. All tests were two-sided, and *p* < 0.05 were considered statistically significant. Statistical tests were performed using the Statistical Package for Social Sciences (version 22.0; SPSS, Chicago, IL, USA).

## 3. Results

Sixty subjects were enrolled in the study: 25 patients with AECOPD, 24 patients with ADHF, and 11 healthy subjects. The clinical features of enrolled subjects are reported in Table 1.

Patients with AECOPD were more frequently men (*p* = 0.088), with a lower body-mass-index and, as expected, were more frequently current or former smokers than patients with ADHF (*p* < 0.001). Patients with AECOPD had a severe functional impairment, assessed from spirometric values obtained in the previous year and collected through their medical record, and the median value of FEV_1_ in patients with COPD was 20% of predicted value (IQR: 25–47), and the median value of FVC was 53% of predicted value (IQR: 45–77). As showed in Table 2, patients with AECOPD presented at ED with more cough and increased sputum volume and purulence than patients with ADHF, while their pulmonary evaluation was characterized by globally diminished vesicular sounds, in contrast with patients with ADHF (*p* < 0.001), and the presence of crackles (*p* < 0.001). Vital signs at ED presentation did not differ between two groups.

### 3.1. Blood Gas Analysis, Blood Tests, Symptoms, and Physical Examination

As shown in Table 3, patients with ADHF have lower hemoglobin, and higher creatinine serum level than patients with AECOPD. N-terminal prohormone of brain natriuretic peptide (NTproBNP) blood levels were lower in patients with AECOPD than in patients with ADHF (*p* < 0.001). Blood gas analysis did not differ between ADHF and AECOPD except for bicarbonate level, lower in patients with ADHF than in patients with AECOPD (*p* = 0.014).

### 3.2. FOT Results

**Figure 1:**
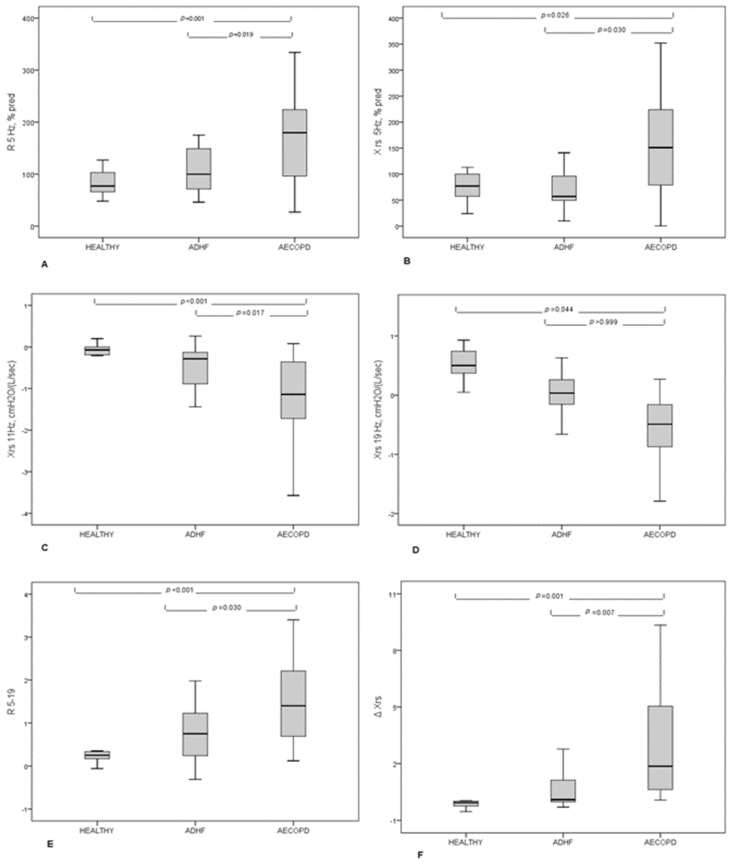
FOT results in patients with AECOPD, ADHF, and healthy subjects. (**A**) Resistance measured at 5 Hz expressed as percentage of predicted value; (**B**) Reactance measured at 5 Hz, expressed as a percentage of predicted values; (**C**) Reactance measured at 11 Hz; (**D**) Reactance measured at 19 Hz; (**E**) Difference between resistance measured at 5 Hz and 19 Hz; (**F**) Mean inspiratory minus mean expiratory reactance expressed as ΔXrs.

As reported in Table 4 and Figure 1, FOT findings differed between patients in terms of inspiratory reactance measured at 5 Hz (Rrs 5 Hz) expressed as percentage of predicted value (% pred), higher in AECOPD than in patients with ADHF, and in terms of inspiratory reactance measured at 5 Hz (Xrs_5_) expressed as percentage of predicted value, lower in patients with AECOPD than in patients with AHF. The difference between Rrs at 5 and at 19 Hz (Rrs_5–19_) was higher in AECOPD than in patients with ADHF (*p* = 0.03). Finally, the expiratory flow limitation (EFL) was significantly higher in patients with AECOPD than in patients with ADHF (*p* = 0.03). ROC-curve analysis of the most clinically significant variables (Rrs Hz %pred, Xrs 5 Hx %pred, Rrs5–19, and a percentual number of flow-limited breaths) was carried on, with Xrs Hz % resulting in the most performant parameter. A value higher than 67% resulted in a ROC AUC for AECB diagnosis of 0.750, with a sensibility of 80% and a sensitivity of 58% (data not shown for the other variables).

### 3.3. Safety

All patients, both from AECOPD and ADHF groups were able to perform bedside FOT measurement despite their clinical conditions. After ED assessment, all patients were then admitted to cardiology, pulmonology, or internal medicine department, and none of these patients were admitted to a sub-intensive care or an intensive care unit.

## 4. Discussion

This is, to our knowledge, the first study evaluating the use of FOT and its results in adult patients presenting with dyspnea in an acute setting. The most important results of our study are: (1) All enrolled patients were able to correctly perform bedside measurement of FOT; (2) Patients with AECOPD show more expiratory flow-limitation than patients with ADHF; (3) The analysis of low FOT frequencies (inspiratory resistance and reactance at 5 Hz), sensitive of pathological structural alteration of the bronchial tree, significantly differs between patients with AECOPD and patients with ADHF.

The misdiagnosis of ADHF in ED is frequent, as demonstrated in a study by Collins et al. [22] in which clinical records of patients with suspected heart failure were evaluated retrospectively. The overall discordance between ED diagnoses and the retrospective diagnosis, established based on standardized criteria, was 14%. Patients with misdiagnosis of ADHF were more likely to have a history of COPD.

To date, many studies were performed to find a valid tool to discriminate between the respiratory or cardiac origin of dyspnea in the ED setting. Lung ultrasound (LUS) has been demonstrated to be a promising instrument in this scenario. A study by Pivetta et al. demonstrated in 2015 that bedside LUS, in addition to the patient clinical evaluation, could improve the accuracy of ADHF diagnosis, in an ED setting [23]. In a small study by Mantuani et al., lung, heart and vena cava bedside US evaluation was proved to be useful in order to immediately exclude ADHF as a diagnostic hypothesis [2]. Nevertheless, lung ultrasound is subject to some limitations. Indeed, this technique needs training to be correctly performed and some reports have shown that patient positioning may impact the number of B-lines in a heart failure population [24].

Spirometry is the main instrumental test to diagnose and assess the response to interventions in COPD [25]. Although, its use in an acute setting remains controversial. Actually, performing a simple spirometry requires a skilled operator and a compliant patient. Giner et al. demonstrated how, even in a routine clinical practice environment and in optimal technical conditions, 15% of the patients could not meet the ATS/ERS 2005 acceptability criteria for simple spirometry [26]. Nevertheless, in a few studies, the authors managed to obtain good data from acceptable maneuvers in the ED, in COPD and asthmatic patients, although they employed operators already trained specifically to take part in clinical studies on asthma [27,28,29]. This approach does not seem to be applicable on a large scale, even though demonstrating that obtaining a spirometry in this setting could be technically possible. On the other hand, FOT has always proved to be a particularly suitable tool for the assessment of mechanical properties of the respiratory system in patients from a different setting with reduced cooperation, though some training for both operator and patient is required. Anyhow, our data confirm this even in a group of patients experiencing an acute decompensation of their chronic diseases.

Tse et al. tested FOT and spirometry in order to investigate the correlation between FOT and spirometric parameters in a geriatric population. The authors described the essential role of FOT measurements, especially in the assessment of the severity of airflow limitation in elderly patients, who showed less cooperation to a forced maneuver [29]. Moreover, FOT is considered particularly suitable for obtaining functional data from children even in acute conditions, as Ducharme et al. demonstrated in a cohort of untrained for forced oscillation children aged 3 to 17 admitted to the ED, assessing changes after treatment during an acute exacerbation of asthma [30].

Although every patient eventually met acceptability criteria for FOT, we did not record the time needed to obtain the measure, which may be longer in this setting, both for difficulties in obtaining an acceptable measure and for the longer time taken into account for recover between measurements in these patients.

Our study showed how FOT maintained the ability to detect flow limitation, which is a hallmark of COPD, both in terms of higher values of the ΔXrs_5_ parameter and in the number of expiratory flow limited breaths in COPD patients, while showing this to be almost absent in ADHF. The ability to identify a pattern clearly relatable to COPD in spite of ADHF using simple spirometry was not found to be true in literature: while in stable conditions, a restrictive pattern can be mostly found in heart failure patients, during the acute phase an obstructive pattern can be due to the compression on the airways generated by interstitial and submucosal edema, possibly added to muscular hyperresponsiveness, and thus leading to misdiagnosis [31,32,33].

In our study, FOT was able to identify more in-depth distinct patterns according to the disease. Firstly, COPD patients showed higher values of frequency-dependent resistance R_5–19_: this parameter was found to be related to an uneven distribution of ventilation both in computational and clinical models [34,35]. This finding during an acute exacerbation confirms what was already found in COPD patients in a condition of stability [35], and the link of these parameters to the possible heterogeneity of the alterations of the airways related to the disease. Regarding ADHF patients, in our study, FOT confirmed previous findings on mechanical properties both in compensated heart failure patients [7] and in an animal model during the development of interstitial edema [18] these patients showed a decrease in the reactance of the airways, as reflected by statistically significant lower values of inspiratory Xrs at 5 Hz. Nonetheless, COPD patients could be identified by greater values of airways resistance evaluated with a frequency resonance of 5 Hz during inspiration, which is a distinctive sign of obstructive disease [21], compared to ADHF. Although the cited studies by Dellacà et al. [18] and Witte et al. [7] showed that resistance could be increased in the heart failure scenario, our data suggests how this property of the lungs is more marked in COPD. Notably, our findings confirm the prominent role of evaluating patients, especially at a frequency of 5 Hz even in this acute setting, reflecting the mechanical properties of the whole airway tree, including peripheral airways, as suggested by the European Respiratory Society task force on FOT [20].

It should be noted that the different regular treatments of the stable disease and the treatments initiated both at home and at the ED for the exacerbation could have influenced to some degree the results at FOT. However, given the population in the study, which included patients with acute deterioration of their chronic disease requiring only an increase of pharmacological regimen and low flow oxygen, some additional consideration must be made: as COPD is by definition characterized by a fixed bronchial obstruction and drugs cannot normalize this feature, the therapy initiated at the ED might have made us lose sensibility in detecting altered mechanical characteristics, and it is plausible that our results would have been even more significant in terms of resistance and reactance and flow limitations when adjusted for therapy. On the other hand, we did not include patients with acute pulmonary edema, as well as none of our patients needed CPAP, which could have rapidly modified pulmonary mechanics along with high doses of diuretics. ADHF patients enrolled in the study certainly needed an increase in dosage of diuretics, but we anticipated that this would have a relatively slow effect on pulmonary mechanics. Since FOT measurements were performed in the first hours of ED admission, we concluded that the effect on the mechanics was still negligible at the time of FOT measurement.

Some limitations of this study deserve discussion. Firstly, the patients enrolled in this study do not represent the full spectrum population of patients admitted at ED for dyspnea. Indeed, we studied a population representing a “pure model” of cardiac decompensation, as reflected by clinical history, physical examination, imaging, and blood tests, by excluding patients with a previous diagnosis of COPD. Moreover, all patients were known and followed previously to the ED admission. Vice versa, we excluded patients with AECOPD who previously received a diagnosis of heart failure or have a cardiologic history. As previously stated, COPD and chronic heart failure can frequently coexist in the same patients. A future devolvement of this study should include more overlapped patients, in order to extend our findings to a more heterogeneous population. Further developments should aim at exploring the usefulness of FOT in terms of predicting prognosis and influencing treatments.

## 5. Conclusions

In conclusion, FOT proved to be a feasible tool to be employed in the ED setting and maintained the ability to identify distinct patterns of mechanical properties of the lung, respectively, relatable to AECOPD or ADHF.

## Figures and Tables

**Table 1:** Characteristics of enrolled patients and healthy subjects.

	AECOPD (*n* = 25)	ADHF (*n* = 24)	HEALTHY (*n* = 11)	*p* *	P AECOPD vs. ADHF **
Age, median IQR, years	70 (66–76)	80 (67–83)	70 (69–71)	0.081	
Male, *n* (%)	20 (80)	12 (50)	7 (64)	0.088	
BMI, kg/m^2^	24.6 (22–28.3)	28.0 (24.5–33.1)	23.1 (22.8–26.6)	**0.017**	**0.049**
Smoking history					
Current, *n* (%)	12 (57)	1 (5)	0 (0)	**<0.001**	**<0.001**
Ex, *n* (%)	9 (43)	9 (43)	1 (9)		
Never, *n* (%)	0 (0)	11 (52)	0 (0)		
Previous AHF, *n* (%)	0 (0)	9 (38)	0 (0)	**<0.001**	**0.001**
Valvular heart disease, *n* (%)	2 (8)	5 (21)	0 (0)	0.154	0.247
OSAS, *n* (%)	1 (4)	2 (8)	0 (0)	0.811	
Atrial fibrillation, *n* (%)	3 (12)	10 (42)	1 (9)	**0.023**	**0.025**
Arterial hypertension, *n* (%)	13 (52)	16 (67)	2 (18)	**0.029**	0.387
Ischemic heart disease, *n* (%)	7 (28)	10 (42)	0 (0)	**0.040**	0.377
Cancer, *n* (%)	2 (8)	2 (8)	0 (0)	0.617	>0.999

AHF, acute heart failure; OSAS, obstructive sleep apnea syndrome * *p* for continuous variables ANOVA or Kruskal–Wallis as appropriate; ** *p* refers to pairwise comparison between AECOPD and ADHF groups. *p* < 0.05 in bold.

**Table 2:** Symptoms and physical examination at presentation at emergency department.

	AECOPD (*n* = 25)	ADHF (*n* = 24)	*p*
Dyspnea			<0.001
new onset, *n* (%)	2 (8)	14 (58)	
worsening from usual, *n* (%)	23 (92)	10 (42)	
Orthopnea, *n* (%)	2 (8)	12 (50)	**0.001**
Cough, *n* (%)	19 (76)	6 (25)	**0.001**
Increased sputum volume, *n* (%)	15 (60)	3 (12)	**0.001**
Increased sputum purulence, *n* (%)	6 (24)	0 (0)	**0.022**
Hemoptysis, *n* (%)	1 (4)	0 (0)	>0.999
Thoracic pain, *n* (%)	3 (12)	2 (8)	>0.999
Fever, *n* (%)	7 (28)	3 (13)	0.289
Oliguria, *n* (%)	0 (0)	4 (17)	0.109
Chest examination			
Normal, *n* (%)	1 (5)	7 (32)	**>0.001**
Globally reduced, *n* (%)	18 (90)	5 (23)	
Focally reduced, *n* (%)	1 (5)	10 (46)	
Rhonchi, *n* (%)	15 (68)	0 (0)	**<0.001**
Crackles, *n* (%)	1 (5)	15 (75)	
Rhonchi and crackles	1 (4)	2 (8)	
Normal heart sound, *n* (%)	17 (85)	9 (41)	**0.005**
Arrhythmia, *n* (%)	1 (5)	7 (32)	**0.047**
Lower limbs swelling, *n* (%)	1 (5)	13 (50)	**<0.001**
Jugular turgescence, *n* (%)	0 (0)	2 (9)	0.305
RR, breath/min	16 (15–19)	17 (15–20)	0.782
HR, beats/min	80 (74–92)	79 (71–93)	0.723
Systolic arterial pressure, mmHg	140 (120–150)	140 (122–155)	0.668
Diastolic arterial pressure, mmHg	80 (70–85)	80 (65–90)	0.957
Body temperature, °C	36.0 (36.0–36.4)	36.0 (36.0–36.0)	0.408
Oxygen therapy, l/min	3 (1–4)	3 (1–3)	0.605

RR: respiratory rate; HR: heart rate. Data are expressed as median and interquartile range. *p* > 0.05 in bold.

**Table 3:** Blood gas analysis and biochemistry in the emergency room.

	AECOPD (*n* = 25)	ADHF (*n* = 24)	*p*
WBC, cells/L	10,200 (8500–12,500)	8800 (8200–11,200)	0.299
Platelets,·cells/L	203,000 (173,000–285,000)	256,000 (175,000–309,000)	0.664
Hemoglobin, g/dL	13.1 (12.0–13.7)	11.0 (10.0–13.7)	**0.018**
Hematocrit, %	38.7 (34.4–42.6)	34.65 (31.3–40.8)	**0.045**
Creatinine, mg/dL	0.9 (0.62–0.9)	1.25 (0.8–1.7)	**0.003**
Azotemia, mg/dL	19 (14–22)	31 (23–39)	**0.001**
Sodium, mEq/L	139 (137–142)	141 (137–143)	0.312
Potassium, mEq/L	4.2 (3.9–4.5)	4.2 (3.9–4.9)	0.609
NTproBNP, pg/mL	435 (179–800)	3965 (1093–5965)	**<0.001**
AST, U/L	20 (17–26)	31 (21–36)	**0.016**
pH *	7.41 (7.38–7.43)	7.40 (7.31–7.42)	0.349
PaO_2_, mmHg	56 (49–59)	55 (49–59)	0.817
PaCO_2_, mmHg	43 (36–48)	38 (34–39)	0.076
HCO_3_, mmol/L	25.4 (24–27)	21.8 (19.7–23.1)	**0.014**

WBC, white blood cells; NTproBNP, N-terminal prohormone of brain natriuretic peptide; AST, aspartate aminotransferase; PaO2, partial pressure of oxygen; PaCO2, partial pressure of carbon dioxide in arterial blood; HCO_3_, bicarbonate; *, arterial hemogasanalysis performed while breathing room air. Data are expressed as median and interquartile range. *p* < 0.05 in bold.

**Table 4:** FOT results.

	AECOPD (*n* = 25)	ADHF (*n* = 24)	HEALTHY (*n* = 11)	*p* *	*p* AECOPD vs. AHF **
Rrs 5 Hz ^†^	4.44 (2.9–5.47)	3.32 (2.25–4.76)	2.12 (1.51–2.39)	**0.002**	0.237
Rrs 5 Hz, %	179 (94–224)	100 (67–149)	77 (63–104)	**0.001**	**0.019**
Xrs 5 Hz ^†^	−1.8 (−2.6–−0.92)	−0.98 (−1.47–−0.53)	−0.77 (−0.97–−0.69)	**0.026**	0.126
Xrs 5 Hz, %	151 (74–231)	57 (49.2–99)	77 (39–102)	**0.005**	**0.005**
Rrs 11 Hz ^†^	3.36 (2.55–4.12)	3.35 (2.04–4.44)	1.99 (1.49–2.42)	**0.010**	>0.999
Rrs 11 Hz, %	141 (93–172)	107 (69–128)	75 (60–104)	**0.008**	0.134
Xrs 11 Hz ^†^	−1.14 (1.73–−0.34)	−0.28 (−0.89–−0.11)	−0.07 (−0.21–0.01)	**<0.001**	**0.017**
Xrs 11 Hz, %	218 (−432–1195)	104 (−33–260)	23 (−97–76)	0.227	
Rrs 19 Hz ^†^	2.76 (2.10–3.31)	2.94 (1.90–3.73)	1.99 (1.19–2.21)	**0.044**	>0.999
Rrs 19 Hz, %	104 (77–132)	88 (62–117)	80 (47–91)	**0.032**	0.424
Xrs 19 Hz ^†^	−0.49 (−0.87–−0.13)	0.03 (−0.17–0.26)	0.50 (0.33–0.78)	**<0.001**	**0.005**
Rrs_5–19_	1.49 (1.03–2.16)	0.44 (0.22–0.76)	0.25 (0.16–0.35)	**<0.001**	**0.030**
ΔXrs_5_^†^	1.86 (0.43–5.14)	0.09 (−0.04–1.16)	−0.70 (−0.26–0.04)	**<0.001**	**0.007**
Flow limitation, %	10 (0–100)	0 (0–12)	0	**<0.001**	**0.030**

Data are expressed as median and interquartile range; For functional data: %, percentage of predicted values; Rrs, resistance; Xrs, reactance; Rrs_5–19_, difference between Rrs5 and Rrs19; ΔXrs, mean inspiratory minus mean expiratory Xrs. Flow limitation is defined as the percentage of total breath with ΔXrs >2.8 cmH_2_O/(L/s) [20]; Rrs and Xrs reported values refer to inspiratory resistance and reactance at 5, 11, and 19 Hz; ^†^: data expressed as cmH_2_O/(L/sec). Functional values for COPD patients are reported as post bronchodilatator therapy; * *p* ANOVA or Kruskal–Wallis as appropriate; ** *p* refers to pairwise comparison between AECOPD and ADHF groups; *p* < 0.05 in bold.

## Data Availability

The data presented in this study are available on reasonable request from the corresponding author.
